# Plant Pathogenic Fungi Special Issue: Genetics and Genomics

**DOI:** 10.3390/microorganisms13040925

**Published:** 2025-04-17

**Authors:** Micael F. M. Gonçalves

**Affiliations:** CESAM (Centre for Environmental and Marine Studies), Department of Biology, Campus Universitário de Santiago, 3810-193 Aveiro, Portugal; micael.goncalves@fc.up.pt

## 1. Introduction

Plant pathogenic fungi pose a significant challenge to global agriculture, threatening crop yields, food security, and ecosystem stability [1]. Over the past decades, advancements in genetics and genomics have revolutionized the study of these fungi, enabling researchers to uncover the molecular basis of pathogenicity, host specificity, and disease progression. High-throughput sequencing technologies, population genomics, and functional genetic studies have facilitated a deeper understanding of fungal evolution, adaptation, and virulence mechanisms [2]. By integrating these approaches, researchers can identify genetic determinants of pathogenicity, track the spread of emerging fungal threats, and develop innovative disease management strategies.

This Special Issue, Plant Pathogenic Fungi: Genetics and Genomics, presents seven studies that contribute to our understanding of fungal taxonomy, phylogenetics, host–pathogen interactions, and disease management. These papers exemplify the power of molecular tools in elucidating fungal diversity, pathogen identification, and disease control strategies. Their collective insights enhance our ability to develop targeted interventions and sustainable management approaches against fungal diseases affecting global agriculture. The integration of genomics with traditional mycology and plant pathology offers a comprehensive framework to tackle emerging fungal threats, improve diagnostic accuracy, and advance breeding programs for resistant crops.

## 2. Advances in Taxonomy and Species Delimitation

The accurate identification of plant pathogenic fungi is critical for effective disease management. Precise species delimitation ensures the correct classification of pathogens, which is fundamental for epidemiological studies and the development of targeted control strategies [3]. Advances in molecular techniques, such as multilocus phylogenetics and coalescent-based analyses, have significantly improved species identification, allowing researchers to distinguish cryptic species and reassess previously misclassified taxa [4]. However, challenges remain due to the inherent complexity of fungal genomes and the frequent occurrence of phenotypic plasticity among closely related species. Pereira et al. (contribution 1) highlight the challenges associated with species delimitation in *Diaporthe* due to cryptic diversification and phenotypic plasticity. By employing an integrative taxonomic approach, including genealogical concordance and coalescent-based species delimitation models, they provide robust evidence that many previously described species within the *Diaporthe arecae* complex are conspecific. Their study underscores the necessity of critical analyses of gene genealogies before describing new fungal taxa and advocates for coalescent methods in multilocus phylogenetic studies. Furthermore, they emphasize the importance of selecting optimal loci for species identification to prevent taxonomic overestimations.

Blanco-Meneses et al. (contribution 2) focus on *Fusarium oxysporum* f. sp. *apii* (Foa) affecting celery in Costa Rica. Using multigene phylogenetics, they identify haplotypes closely related to Foa race 3 (which is virulent only to green celery), demonstrating high virulence on imported commercial celery cultivars. Their findings highlight the lack of fully resistant cultivars against this pathogen and emphasize the urgent need for country-specific guidelines to prevent the introduction of new *Fusarium* races. By providing crucial insights into host susceptibility, this study underscores the importance of disease monitoring and resistant cultivar trials to safeguard celery production.

## 3. Host–Pathogen Interactions and Disease Ecology

The interactions between fungal pathogens and host plants play a crucial role in disease development. Understanding these interactions is fundamental for designing effective disease management strategies, including the deployment of resistant crop varieties and the implementation of integrated pest management approaches. Recent studies have highlighted the role of fungal secondary metabolites, effector proteins, and host immune responses in shaping disease outcomes.

Fuentes et al. (contribution 3) explore alternative management strategies for *Pythium amazonianum*, a recently identified causal agent of avocado tree wilt. Their study demonstrates the antifungal efficacy of extracts from *Proboscidea parviflora* and *Phaseolus lunatus*, suggesting that these natural compounds could serve as sustainable alternatives to synthetic fungicides. Notably, their findings indicate differential inhibitory effects and lethal time responses depending on extract concentration and fungal strain, reinforcing the need for further evaluation across diverse phytopathogenic fungi and oomycetes.

Solano-Báez et al. (contribution 4) document the first report of *Alternaria alternata* and *A. gossypina* causing leaf blight in *Castilleja tenuiflora*, a medicinal plant endemic to Mexico. Their work highlights the potential threat of these pathogens to native populations and underscores the necessity of in vitro propagation strategies for conservation. Given the pharmacological significance of *C. tenuiflora*, this study raises concerns about the broader ecological and economic implications of fungal infections in wild medicinal plants and calls for comprehensive monitoring of *Alternaria* spp. in forest ecosystems.

Schierling et al. (contribution 5) describe *Neopestalotiopsis rosae* as an emerging pathogen responsible for leaf blight and fruit rot in strawberries in Germany. Through morphological, molecular, and pathogenicity analyses, they provide the first documented evidence of *N. rosae* in Europe. Their study calls for proactive disease monitoring and management to mitigate economic losses in strawberry production. The virulence observed across various cultivars, coupled with environmental influences on disease severity, underscores the importance of integrating molecular diagnostics with field surveillance to contain pathogen spread and protect commercial strawberry production.

## 4. Molecular Mechanisms of Pathogenicity

Understanding the genetic mechanisms underlying fungal virulence is essential for developing effective disease control strategies. Pathogenic fungi deploy an array of virulence factors, including toxins, effector proteins, and enzymatic arsenals that facilitate host colonization [5]. Genetic and functional studies of these virulence determinants offer valuable insights into fungal adaptation and host specificity, paving the way for targeted interventions such as RNA interference-based fungicides, gene editing, and resistance breeding programs.

Fu et al. (contribution 6) investigate the role of the cytochrome P450 monooxygenase *Aacp1* in *Alternaria alternata*, revealing its involvement in sporulation, oxidative stress response, ACT toxin production, and pathogenicity. Additionally, their findings suggest a novel regulatory function of *Aacp1* on *AaNdo1*, a gene linked to sporulation. This study contributes to a deeper understanding of cytochrome P450 monooxygenases and their roles in fungal development and pathogenicity, providing a potential target for disease intervention strategies.

Luong et al. (contribution 7) assess the genetic diversity and virulence of *Phytophthora sojae* isolates in the Republic of Korea. Using molecular markers and phenotypic assays, they demonstrate high pathotype complexity among six isolates. While the sample size is small, their findings provide a foundation for future large-scale studies on *P. sojae* diversity and inform soybean breeding programs for improved resistance. This research underscores the necessity of long-term, nationwide monitoring using time-series data to track pathotype evolution and enhance resistance breeding efforts for sustainable soybean cultivation.

## 5. Conclusions

The studies presented in this Special Issue showcase the critical role of genetics and genomics in advancing fungal pathogen research. From refining species delimitation to elucidating host–pathogen interactions and uncovering molecular mechanisms of pathogenicity, these contributions pave the way for improved disease management strategies. Future research should continue integrating phylogenetics, population genetics, and molecular biology to address emerging challenges in plant pathology. The ongoing evolution of plant pathogenic fungi necessitates continuous monitoring and innovation in disease control methodologies to safeguard global agriculture and biodiversity. By leveraging cutting-edge genetic tools and interdisciplinary approaches, researchers can develop sustainable solutions to mitigate the impact of fungal diseases on food security and ecosystem health.
